# The ssDNA-binding protein MEIOB sustains synaptonemal complex integrity by stabilizing recombination intermediates during male meiosis

**DOI:** 10.1016/j.isci.2026.118103

**Published:** 2026-11-02

**Authors:** Haiwei Feng, Binjie Jiang, Yanling Gao, Muhammad Zubair, Li Hu, Hongling Wang, Xuemei Wang, Miao Li, Mengcheng Luo, Jingxia Sun, Lijun Huo, Dongteng Liu

**Affiliations:** 1 Department of Reproductive Health and Infertility, South China Hospital, Medical School, Shenzhen University, Shenzhen 518116, China; 2 Guangdong Key Laboratory for Biomedical Measurements and Ultrasound Imaging, National-Regional Key Technology Engineering Laboratory for Medical Ultrasound, School of Biomedical Engineering, Shenzhen University Medical School, Shenzhen 518060, China; 3 Guangdong Key Laboratory of Urology, Department of Urology, The First Affiliated Hospital of Guangzhou Medical University, Guangzhou, Guangdong 510000, China; 4 Shenzhen Key Laboratory of Fertility Regulation, Reproductive Medicine Center, The University of Hong Kong-Shenzhen Hospital, Shenzhen 518053, China; 5 Department of Obstetrics and Gynecology, Li Ka Shing Faculty of Medicine, The University of Hong Kong, Hong Kong SAR 999077, China; 6 Maternal-Fetal Medicine Institute, Department of Obstetrics and Gynecology, Shenzhen Baoan Women’s and Children’s Hospital, Shenzhen 518100, China; 7 Department of Obstetrics, South China Hospital, Medical School, Shenzhen University, Shenzhen 518116, China; 8 Center for Reproductive Medicine, Shanghai First Maternity and Infant Hospital, School of Medicine, Tongji University, Shanghai 201204, China; 9 Hubei Provincial Key Laboratory of Developmentally Originated Disease, School of Basic Medical Sciences, Wuhan University, Wuhan 430072, China; 10 Biomedical Research Institute, Hubei University of Medicine, Shiyan 442000, China

**Keywords:** synaptonemal complex, meiotic recombination, ZZS complex, MEIOB, premature desynapsis

## Abstract

The synaptonemal complex (SC) is fundamental to meiotic recombination and crossover formation between homologous chromosomes. In yeast, the SC is assembled and sustained by the ZZS complex (Zip2-Zip4-Spo16). However, how ZZS components are recruited and stabilized at chromosomes, and whether their mammalian orthologs (SHOC1-TEX11-SPO16) play similar roles, remain poorly understood. Here, we report that germ cell-specific ablation of the meiosis-specific single-stranded DNA-binding protein MEIOB in mice causes premature chromosomal desynapsis in early pachytene spermatocytes, leading to their elimination before mid-pachytene. Mechanistically, MEIOB associates with SPO16 *in vitro*, and *Spo16*-deficient pachytene-like spermatocytes display comparable premature desynapsis and severely reduced MEIOB foci on chromosomal axes. Moreover, the recruitment of another ZZS component, TEX11, to recombination intermediates is markedly diminished in *Meiob* knockout pachytene spermatocytes. Together, our findings suggest that the MEIOB-SPATA22 complex may cooperate with SPO16 to support SC integrity and crossover formation during mammalian meiosis.

## Highlights

• Inducible *Meiob* deletion in mice causes premature SC disassembly in pachynema
• MEIOB associates with the ZZS component SPO16 on meiotic chromosome axes
• SPO16 loss phenocopies *Meiob*^cKO^ desynapsis and reduces axial MEIOB–SPATA22

## Graphical abstract

**Figure:**
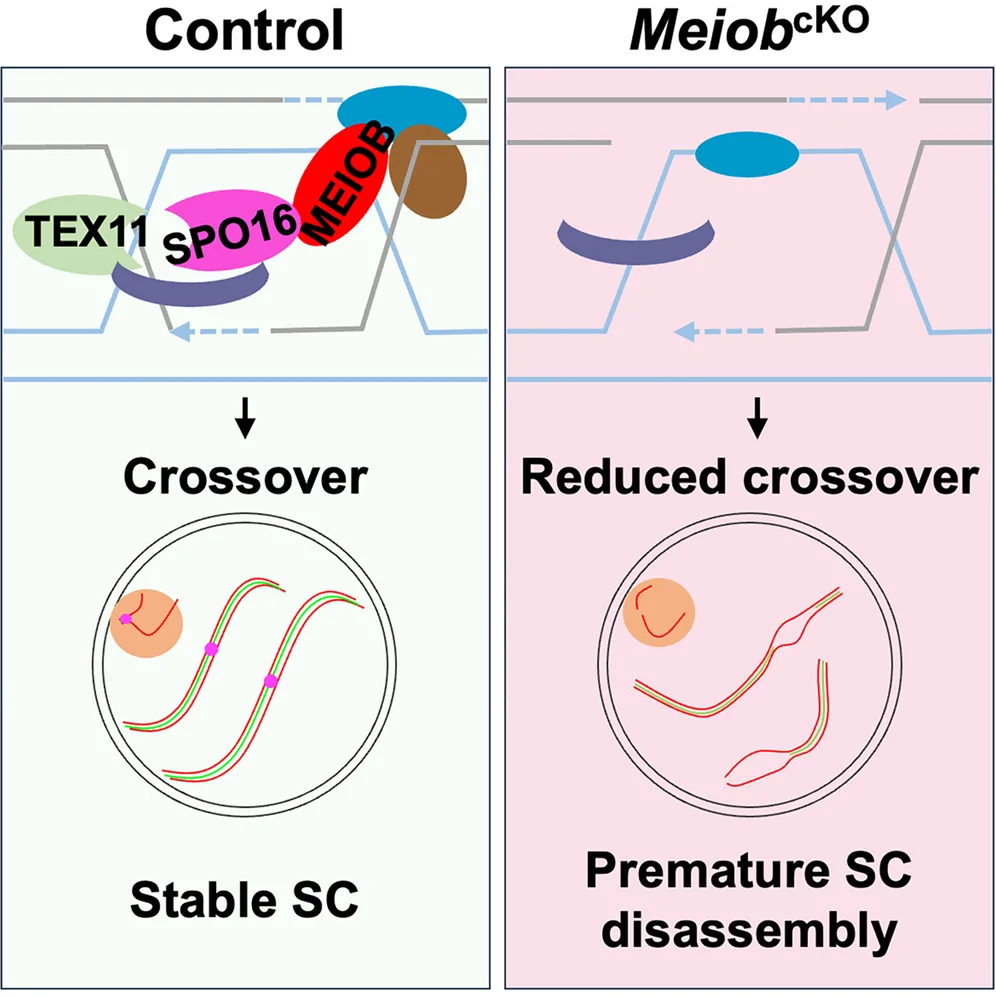

## Teaser

Molecular biology; Cell biology

## Introduction

Meiotic recombination is a mandatory process during the prophase of meiosis I. This process depends on the formation of the synaptonemal complex (SC), a zipper-like polymeric protein structure that connects homologous chromosomes and is evolutionarily conserved across species.^1^^,^^2^ In mice, knockout of SC components disrupts homologous synapsis in meiocytes, impairing double-strand break (DSB) repair and crossover formation.^3^ Although the importance of SC formation in meiotic recombination is well established, the mechanisms that maintain SC stability during pachynema remain poorly understood.

In yeast, plants, and mammals, a conserved family of ZMM proteins facilitates SC formation and crossovers.^4^^,^^5^^,^^6^ In yeast, at least eight ZMM proteins, including Zip1/2/3/4, Mer3, Msh4/5, and Spo16, are involved in this process.^4^^,^^6^ Recent studies in budding yeast have demonstrated that both the specific cleavage of Holliday junctions (HJs) and the conditional degradation of ZMM proteins lead to rapid SC disassembly. This finding underscores the critical role of the continuous presence of ZMM proteins in SC polymerization and maintenance.^7^^,^^8^ Zip2, Zip4, and Spo16 form a functional ZMM subcomplex called ZZS, and disruption of their mammalian orthologs—SHOC1, TEX11, and SPO16—in mice results in defects in SC stability and causes male infertility.^9^^,^^10^^,^^11^^,^^12^^,^^13^

MEIOB, a meiosis-specific protein with an oligonucleotide-binding (OB)-fold domain, forms a stable heterodimeric complex with its binding partner SPATA22 and binds to single-stranded DNA (ssDNA).^14^^,^^15^ Previous studies have reported that MEIOB is required for DSB repair by facilitating the recruitment of the recombinases RAD51 and DMC1 to DSB sites in the early stages of meiosis.^16^^,^^17^^,^^18^^,^^19^ The DSB repair facilitates homology search and promotes the formation of SC and early recombination intermediates. Moreover, analysis of the *Meiob* knock-in mice that mimic a missense mutation in an azoospermic patient reveals that MEIOB acts as a dose-dependent regulator in meiotic recombination.^20^ However, the precise mechanism by which MEIOB mediates crossover regulation remains unclear.

In this study, we generated a germ cell-specific conditional knockout mouse model for the *Meiob* gene (*Meiob*^cKO^). By studying this mouse line, we found that unlike previously reported global *Meiob* knockout models that arrest early due to defective meiotic recombination and homologous synapsis, our inducible *Meiob*^*cKO*^ model uncovers a previously unappreciated late-stage role for MEIOB. Although SC assembly is initially established, loss of MEIOB results in premature desynapsis and destabilization of the SC during pachynema. This function may be regulated by a previously uncharacterized MEIOB-SPO16 interaction that stabilizes the ZZS complex in early recombination intermediates. These findings suggest that MEIOB plays a critical role in maintaining the structural integrity of the SC in pachytene spermatocytes. Thus, our study demonstrates that the MEIOB-SPATA22 complex may serve as a critical link bridging the HJs and ZZS complexes during mammalian meiosis.

## Results

### Germ cell-specific knockout of *Meiob* in mouse spermatocytes

To further study the function of MEIOB in male meiosis, we generated *Meiob*^fl/fl^; *Ddx4-*Cre^ERT2^ mice in which functional MEIOB would be eliminated in germ cells upon tamoxifen induction (Figures 1A and S1A). We first administered two tamoxifen injections (20 mg/kg) to these mice at PD14 and PD15, then collected testes at various days post-treatments (dpt) (Figure 1B), herein referred to as *Meiob* conditional knockout (*Meiob*^cKO^) testes. The *Meiob*^fl/fl^ littermate mice were used as controls. At 7 dpt and 10 dpt, the testes of *Meiob*^cKO^ mice became visibly smaller than those of control mice (Figure 1B). Genotyping and immunofluorescence analyses confirmed the knockout of the *Meiob* gene and successful depletion of the MEIOB protein from chromosome axes in the nuclei of 6 dpt *Meiob*^cKO^ zygotene-like and pachytene-like spermatocytes (Figures 1C and S1B). Similarly, MEIOB-binding partner SPATA22 also disappeared from *Meiob*^cKO^ spermatocytes (Figure S1C). These results suggest that we have successfully obtained *Meiob*^cKO^ spermatocytes.

**Figure 1:**
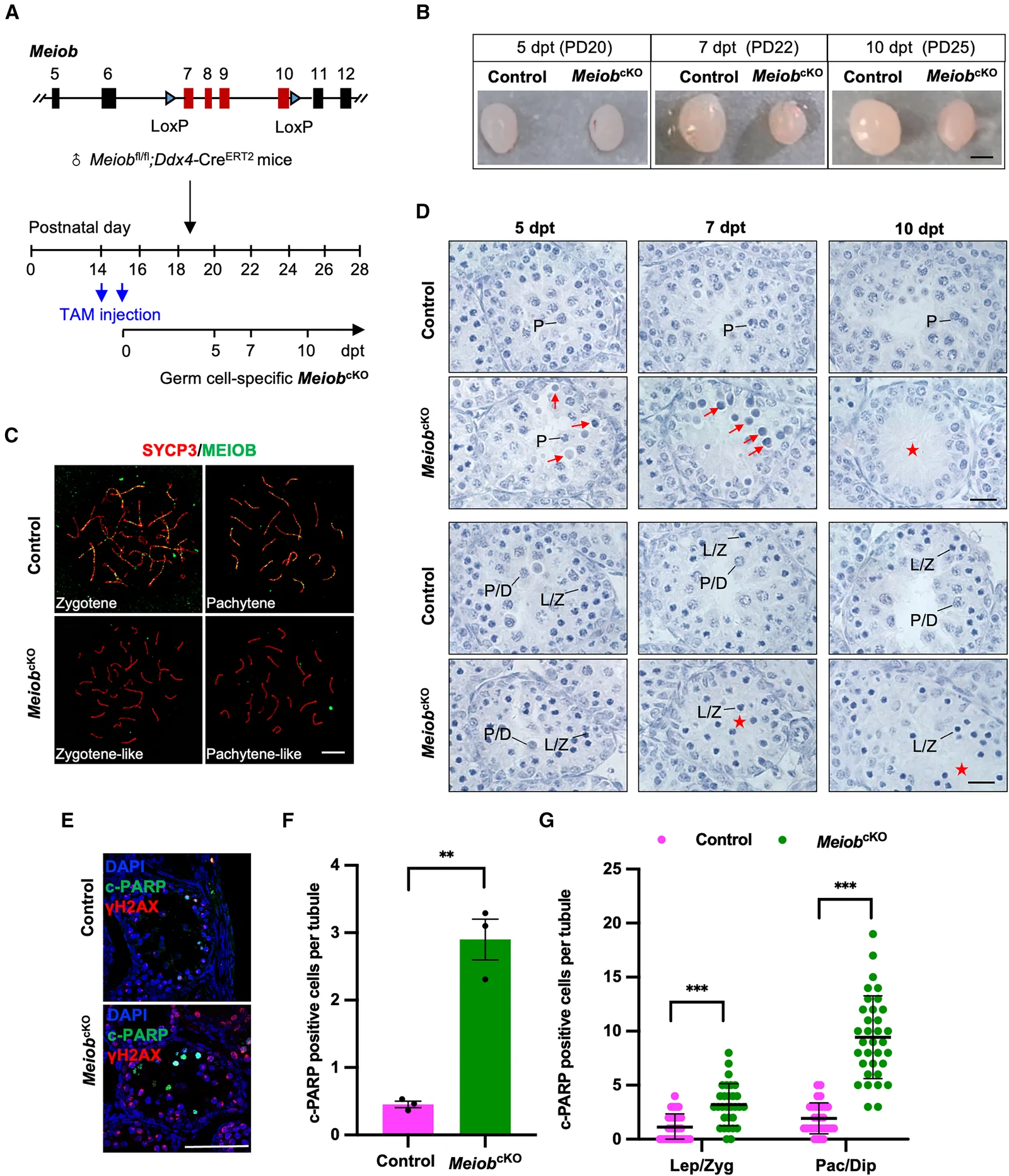
*Meiob*^cKO^ mice exhibit elevated apoptosis in pachytene spermatocytes (A) Schematic diagram of the *Meiob* floxed allele mouse gene, where two *loxP* sites are inserted in the 6th and 10th introns. Tamoxifen-induced *Ddx4*-Cre^ERT2^ strategy to knockout *Meiob*; blue arrows indicate PD14 and PD15 for 2 consecutive daily injections of tamoxifen. (B) Comparison of the testis morphology of mice at different time points after tamoxifen injection. Scale bars, 2 mm. (C) Immunostaining of MEIOB (green) and SYCP3 (red) on spermatocyte chromosome spreads of control and *Meiob*^cKO^ mice. Scale bars, 5 μm. (D) Histological analysis of the mouse testes at different time points after tamoxifen injection. Red arrows indicate apoptotic pachytene spermatocytes; asterisk indicates loss of pachytene or diplotene spermatocytes. L/Z, Leptotene/Zygotene; P, Pachytene; D, Diplotene. Scale bars, 50 μm. (E) Co-immunostaining of c-PARP (green, apoptosis marker) and γH2AX (red, meiotic staging marker) on testis sections from control and *Meiob*^cKO^ mice. DNA was stained with DAPI (blue). Scale bars, 50 μm. (F) Numbers of c-PARP-positive cells per tubule. The mean was calculated for each male, and data from three males for each genotype were used. At least 30 tubules for each mouse were counted. The data are shown as mean ± S.D. ∗∗*p* < 0.01, two-way ANOVA. (G) To precisely determine the meiotic stages of these dying cells, we utilized distinct γH2AX localization patterns to classify the seminiferous tubules into Leptotene/Zygotene (Lep/Zyg) and Pachytene/Diplotene (Pac/Dip) stages. Quantification of c-PARP^+^ cells across tubules with distinct γH2AX patterns. Data represent the mean calculated from three males per genotype, with at least 30 tubules analyzed per mouse. Data are shown as mean ± S.D. ∗∗∗*p* < 0.001, two-way ANOVA.

### Knockout of *Meiob* induced pachytene spermatocyte apoptosis in mice

Histological analysis of *Meiob*^cKO^ testes revealed that at 5 dpt, pachytene spermatocytes characterized by thick, coarse chromatin threads had begun to undergo apoptosis (Figure 1D, red arrows). By 7 dpt, a significant loss of these pachytene cells was observed, and multiple seminiferous tubules appeared depleted of late-stage spermatocytes (Figure 1D, asterisks). As a result, by 10 dpt, multiple seminiferous tubules lacked spermatocytes or contained only zygotene spermatocytes (Figure 1D).

Immunostaining of 6 dpt testis sections with γH2AX and c-PARP (cleaved poly ADP ribose polymerase), an apoptosis marker, revealed a significant increase in c-PARP-positive cells in *Meiob*^cKO^ seminiferous tubules, including many pachytene-stage tubules (Figures 1E and 1F). Quantitative analysis further showed that, although only a mild increase in apoptosis was observed in Lep/Zyg-stage tubules, c-PARP-positive cells accumulated dramatically in Pac/Dip-stage tubules of *Meiob*^cKO^ mice (Figure 1G). Together, these findings suggest that MEIOB likely plays an important role in the later stage of meiotic prophase Ⅰ.

### *Meiob*^cKO^ pachytene spermatocytes exhibited premature SC disassembly

We looked into the aspect of homologous synapsis in *Meiob*^cKO^ spermatocytes by examining the chromosome spreads immunostained with the SC transverse filament SYCP1 and the lateral element SYCP3. As shown in Figure 2A, two types of pachytene-like spermatocytes were observed in spreads from *Meiob*^cKO^ testis, both of which were absent in the control testis. Pachytene-like 1 spermatocytes exhibited strong SYCP1 signals at the synapsis region of homologous chromosomes (Figure 2A, arrowhead). In contrast, pachytene-like 2 spermatocytes exhibited sharply reduced and discontinuous SYCP1 in many homologous chromosomes (Figure 2A, arrow).

**Figure 2:**
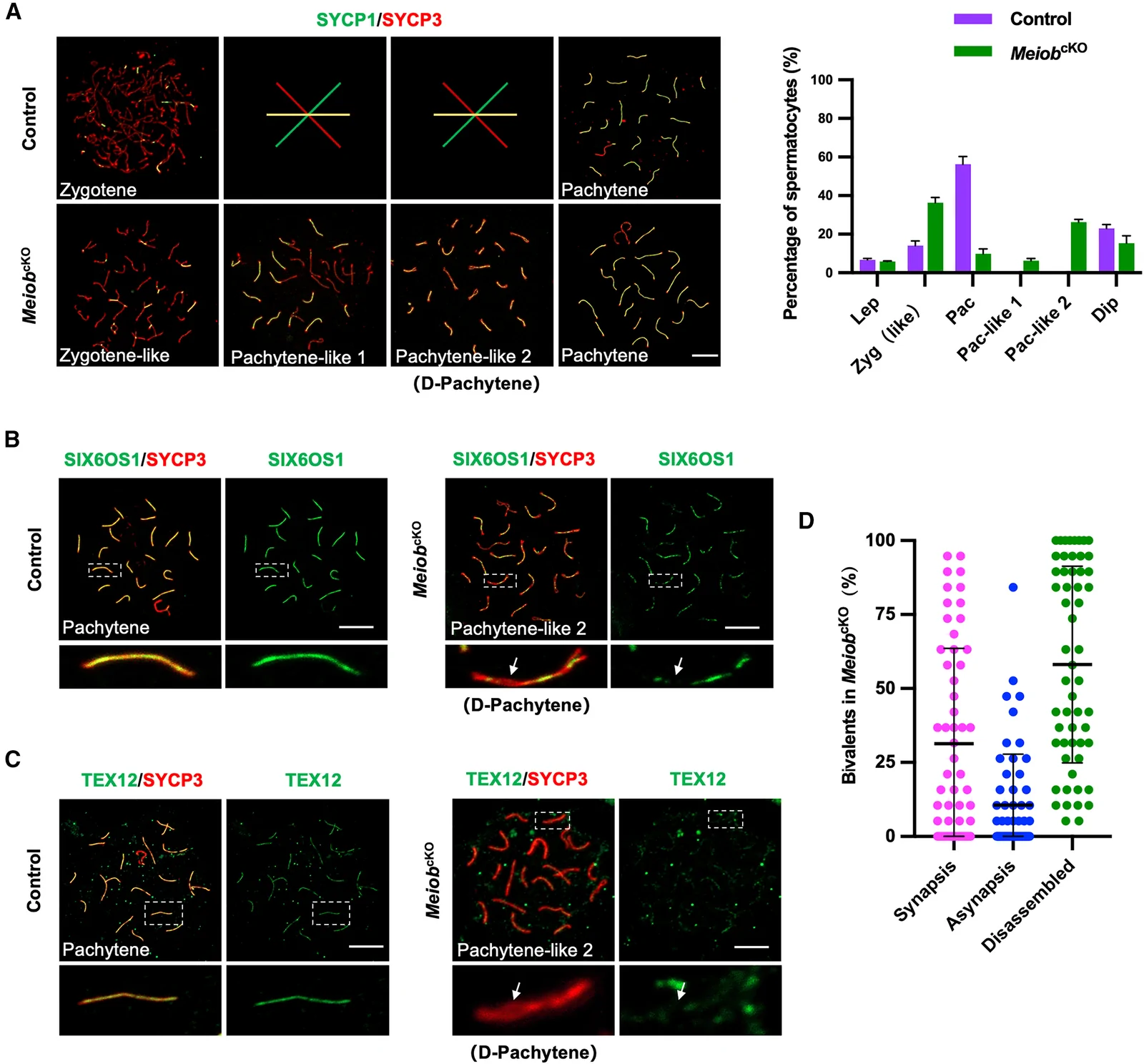
*Meiob*^cKO^ pachytene spermatocytes exhibited synaptic defects (A) Immunostaining of SYCP1 (green) and SYCP3 (red) on spermatocyte chromosome spreads of control and *Meiob*^cKO^ mice. Quantitative analysis of spermatocyte populations in control and *Meiob*^cKO^ testes. The graph represents the mean percentage (mean ± S.D.) of different spermatocyte types, based on 892 control and 860 *Meiob*^cKO^ spermatocytes from 3 mice per genotype, two-way ANOVA. (B) Representative spreads of spermatocytes from control and *Meiob*^cKO^ mice co-stained with SIX6OS1 (green) and SYCP3 (red). Scale bars, 5 μm. Enlarged views of the boxed chromosomes are shown below. (C) Representative spreads of spermatocytes from control and *Meiob*^cKO^ mice co-stained with TEX12 (green) and SYCP3 (red). Scale bars, 5 μm. Enlarged views of the boxed chromosomes are shown below. (D) Quantitative analysis of synaptic defects in *Meiob*^cKO^ D-pachytene spermatocytes. The graph indicates the percentages of cells exhibiting synaptic, asynaptic, or disassembled chromosomes. A total of 59 D-pachytene spermatocytes across three *Meiob*^cKO^ mice were analyzed. Data are presented as mean ± S.D.

By immunostaining for SIX6OS1 and TEX12, two key components of SC central elements, we found that *Meiob*^cKO^ pachytene-like 2 spermatocytes exhibited attenuated SIX6OS1 and TEX12 signals at chromosome axes (Figures 2B and 2C). We also noticed that some autosomal bivalents displayed discontinuous SIX6OS1 and TEX12 at the homologous region (Figures 2B and 2C, arrows). Quantitative analysis showed that in *Meiob*^cKO^ pachytene-like 2 spermatocytes, over 50% of autosomal bivalents showed such synaptic defects (Figure 2D). By classifying *Meiob*^cKO^ pachytene spermatocytes according to chromosomal synapsis defects, we found that the majority (∼60.8%) of *Meiob*^cKO^ pachytene-like spermatocytes exhibited separated sex chromosomes (Figures S2A and S2B). These results indicate that SC stability is compromised in *Meiob*^cKO^ pachytene-like 2 spermatocytes. We referred to such pachytene-like spermatocytes as “D-pachytene” in the following analyses.

To examine if the severely abnormal bivalents observed at later stages in *Meiob*^cKO^ had previously achieved synapsis with normal SC assembly, we assessed the localization dynamics of HORMAD1 and BRCA1, two established markers of unsynapsed chromosome axes. HORMAD1 accumulates on unsynapsed axes and is rapidly evicted from the axes upon homologous synapsis,^21^^,^^22^^,^^23^ whereas BRCA1 localizes specifically to unsynapsed axes during zygonema.^24^^,^^25^ In *Meiob*^cKO^ zygotene-like and pachytene-like 1 spermatocytes, both HORMAD1 and BRCA1 localized normally to unsynapsed chromosome axes (Figures 3A and 3B). Moreover, both proteins were efficiently depleted from synapsed chromosomal regions in *Meiob*^*cKO*^ pachytene-like spermatocytes, similar to control pachytene cells. These observations suggest that SC assembly likely occurs normally at synapsed chromosomal regions during the early stages of *Meiob*^cKO^ spermatocytes.

**Figure 3:**
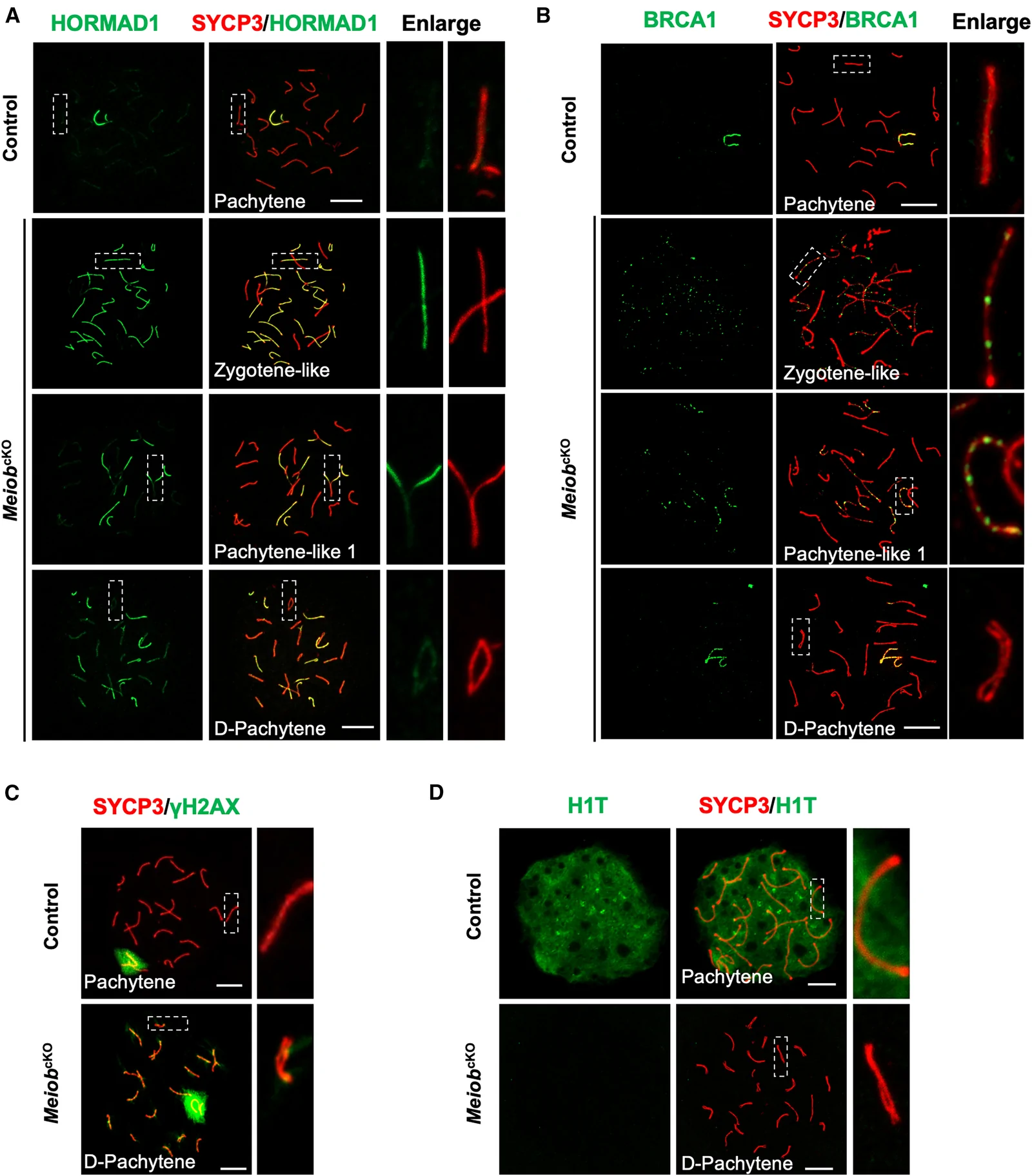
*Meiob*^cKO^ mice exhibited premature SC disassembly in pachytene-like II spermatocytes (A) Immunostaining of BRCA1 (green) and SYCP3 (red) of spermatocytes from control and *Meiob*^cKO^ mice. Scale bars, 5 μm. (B) Immunostaining of HORMAD1 (green) and SYCP3 (red) of spermatocytes from control and *Meiob*^cKO^ mice. An enlarged view of the area within the box shows the autosomes. (C) Immunostaining of γH2AX (green) and SYCP3 (red) of spermatocytes from control and *Meiob*^cKO^ mice. An enlarged view of the area within the box shows the autosomes on the right. Scale bars, 5 μm. (D) Representative spreads of spermatocytes from control and *Meiob*^cKO^ mice co-stained for H1T (green) and SYCP3 (red). An enlarged view of the area within the box shows the autosomal chromosome. Scale bars, 5 μm.

We found that in *Meiob*^cKO^ D-pachytene spermatocytes, HORMAD1 signals largely disappeared not only from synapsed chromosomes but also from the aberrant bivalents exhibiting synaptic defects (Figure 3B). Similarly, these abnormal bivalents in *Meiob*^cKO^ D-pachytene spermatocytes lacked BRCA1 signals on autosomes, resembling fully synapsed normal bivalents observed in wild-type pachytene spermatocytes (Figures 3A and 3B). Together, these findings suggest that knockout of *Meiob* may lead to premature desynapsis, rather than a defect in SC assembly, in homologous bivalents during pachytene. Notably, this premature desynapsis may alter chromosome status in a manner that prevents the reloading of HORMAD1 onto desynapsed chromosomes, as normally occurs during diplonema.^21^^,^^26^

By immunostaining for γH2AX, a marker for DNA damage, we found that compared to the wild-type pachytene spermatocytes, where γH2AX signals were exclusively aggregated at the sex chromosome area, *Meiob*^cKO^ D-pachytene spermatocytes retained aberrant γH2AX signals on autosomes, particularly those with prematurely disassembled SC (Figure 3C). To further analyze the meiotic progression of D-pachytene spermatocytes, chromosome spreads were immunostained for H1T, a mid-to-late pachytene marker. The results showed that *Meiob*^cKO^ D-pachytene spermatocytes were H1T-negative, indicating that D-pachytene spermatocytes may not surpass the mid-pachytene checkpoint and thus undergo apoptosis (Figure 3D).

### MEIOB may interact with SPO16 in preventing premature desynapsis in pachytene spermatocytes

SPO16, together with TEX11 and SHOC1, constitutes the mammalian ZZS complex. Previous studies have shown that SPO16 is required for SC stability in mice,^12^ suggesting a potential role of the ZZS complex in regulating SC integrity. Given that loss of MEIOB caused premature desynapsis, we investigated whether MEIOB interacts with components of the ZZS complex. Thus, we conducted co-immunoprecipitation (Co-IP) experiments in HEK293T cells transfected with pcDNA3.1(+)-3×HA-*Meiob*-GFP, pCAG-3×FLAG-*Spo16*-GFP, pcDNA3.1(+)-3×FLAG-*Shoc1*-GFP, or pcDNA3.1(+)-3×FLAG-Tex11-GFP plasmids. Our results showed that MEIOB was associated with SPO16, but not with SHOC1 or TEX11, under the conditions tested (Figures 4A–4C). DNase treatment of cell lysates did not disrupt the MEIOB–SPO16 association (Figure S3), arguing against a DNA-mediated interaction. Given that SPO16 is required for SC stability in mice,^12^ these findings raise the possibility that MEIOB cooperates with SPO16 in maintaining SC integrity during meiosis.

**Figure 4:**
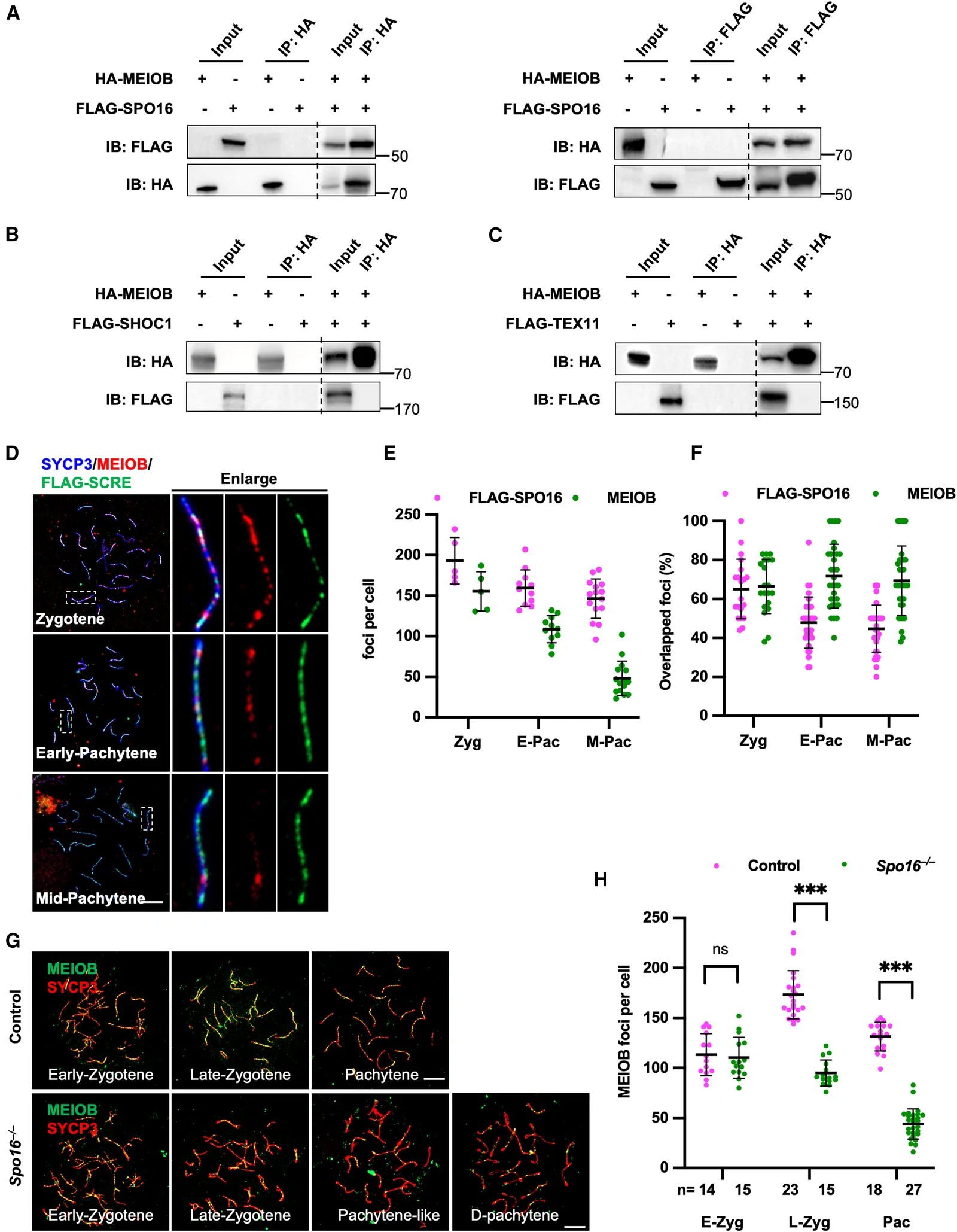
MEIOB interacts with SPO16 to facilitate the recruitment of TEX11 to recombination intermediates (A–C) Co-immunoprecipitation analysis of the MEIOB with SPO16, SHOC1, and TEX11 in HEK293T cells. Cells transfected with each tagged plasmid alone served as negative controls to rule out non-specific binding to the beads, antibodies, or epitope tags. Molecular weight markers (kDa) are indicated. Vertical dashed lines indicate splicing of lanes. (D) Immunostaining of FLAG-SPO16 (green) and MEIOB (red) on spermatocyte chromosome spreads of wild-type testes electroporated with plasmids expressing FLAG-SPO16. An enlarged view of the area within the box shows the autosomes on the right. (E) Paired quantification of SPO16 (magenta) and MEIOB (red) foci within the same spermatocytes from electroporated mice at the indicated meiotic stages (Zyg, E-Pac, and M-Pac). Each dot represents an individual analyzed spermatocyte from the electroporated mice. Spermatocytes were collected from two electroporated mice. Data are shown as mean ± S.D. (F) Quantitative analysis of SPO16 and MEIOB colocalization in spermatocytes. Each dot represents an individual spermatocyte (*n* = 20, 32, and 33 for Zyg, E-Pac, and M-Pac, respectively), collected from two electroporated mice. Data are shown as mean ± S.D. (G and H) Immunostaining of MEIOB (green) and SYCP3 (red) on pachytene spermatocyte chromosome spreads of control and *Spo16*^−/−^ mice (G). The number of MEIOB foci was counted (H). “n” represents the number of spermatocytes analyzed from 3 mice per genotype. ∗∗∗*p* < 0.001, The Mann–Whitney U test was applied to calculate *p* values. Scale bars, 5 μm.

We next examined the spatial relationship between MEIOB and SPO16 in spermatocyte spreads following testicular electroporation with an FLAG*-Spo16* overexpression plasmid. Triple immunostaining of SYCP3, FLAG, and MEIOB revealed that over 60% of SPO16 and MEIOB foci overlapped in late zygotene spermatocytes (Figure 4D). From zygonema to pachynema, while the number of MEIOB foci was strikingly reduced, the number of SPO16 foci remained stable (Figure 4E). The percentage of overlapping MEIOB-SPO16 foci among MEIOB foci remained relatively unchanged (over 60%), whereas the corresponding percentage among SPO16 foci sharply decreased to 45% from zygonema to pachynema (Figure 4F). These observations suggest that MEIOB and SPO16 may function coordinately during meiotic prophase, with MEIOB dissociating from chromosomal sites earlier than SPO16.

We observed that the *Spo16* knockout (*Spo16*^−/−^) testis also contained D-pachytene spermatocytes, which exhibited premature desynapsis in many autosomal bivalents with normal HORMAD1 evacuation (Figure S4). Similar to our *Meiob*^cKO^ model, these *Spo16*^−/−^ D-pachytene spermatocytes were H1T-negative and failed to progress beyond early pachynema (Figure S5). As our western blot and fluorescence analyses have suggested that MEIOB is highly associated with SPO16 in spermatocytes, we hypothesized that loss of MEIOB function may contribute to the premature desynapsis phenotype in *Spo16*^−/−^ D-pachytene spermatocytes. To this end, we quantified MEIOB localization in *Spo16*^−/−^ spermatocytes. We found that the number of MEIOB foci was significantly reduced in *Spo16*^−/−^ D-pachytene spermatocytes compared to controls (Figures 4G and 4H). Similarly, a highly significant reduction was also observed for SPATA22 in the mutant spermatocytes across both zygotene-like and D-pachytene stages (Figures S6A and S6B). These findings suggest that SPO16 is required for proper chromosomal accumulation of the MEIOB-SPATA22 complex during meiotic prophase. Consequently, the disruption of the MEIOB-SPO16 interaction may contribute to the premature desynapsis observed in pachytene spermatocytes.

### MEIOB facilitates the accumulation of ZMM proteins for crossover formation

During meiotic recombination, the ZZS complex component SPO16 coordinates with the MutSγ complex (MSH4-MSH5) and the SUMO ligase RNF212 to promote crossover designation.^27^ To determine whether MEIOB similarly regulates crossover-associated recombination factors, we first examined the chromosomal localization of RNF212 in spermatocytes. In control mice, RNF212 foci normally appeared on the synapsed chromosome axes during zygonema and peaked at early pachynema. However, RNF212 foci were markedly reduced in *Meiob*^cKO^ spermatocytes from the zygotene-like to early pachytene-like stages (Figures 5A and 5B), indicating that the recruitment of RNF212 is severely impaired in the absence of MEIOB.

**Figure 5:**
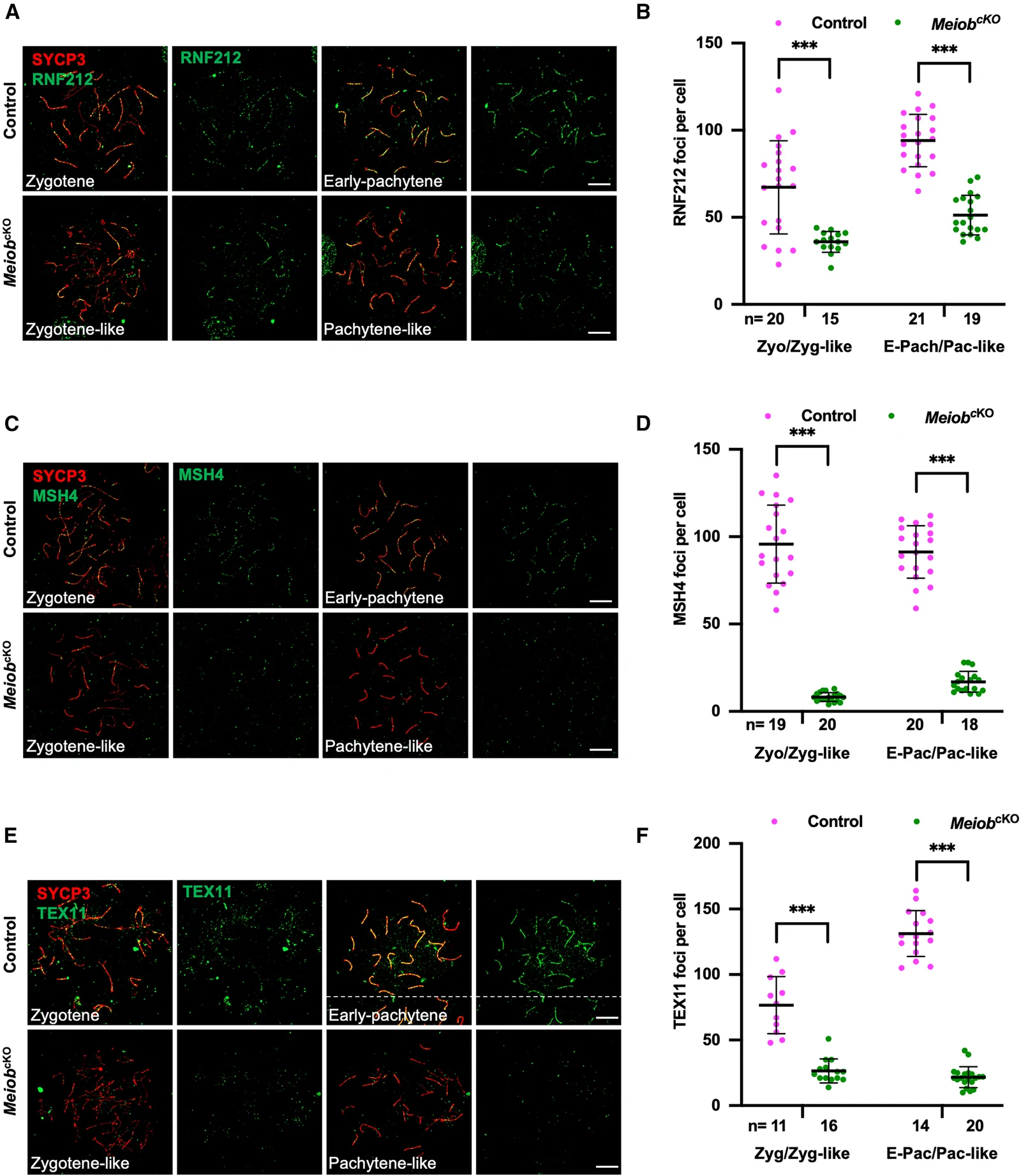
MEIOB is required for the chromosomal accumulation of ZMM proteins during meiotic recombination (A and B) Immunostaining of RNF212 (green) and SYCP3 (red) on zygotene and early-pachytene spermatocyte chromosome spreads of control and *Meiob*^cKO^ mice (A). The number of RNF212 foci was counted (B). “n” represents the number of spermatocytes from 3 mice for each genotype. The data are shown as mean ± S.D. ∗∗∗*p* < 0.001, The Mann–Whitney U test was applied to calculate *p* values. Scale bars, 5 μm. (C and D) Immunostaining of MSH4 (green) and SYCP3 (red) on zygotene and early-pachytene spermatocyte chromosome spreads of control and *Meiob*^cKO^ mice (C). The number of MSH4 foci was counted (D). “*n*” represents the number of spermatocytes from 3 mice for each genotype. The data are shown as mean ± S.D. ∗∗∗*p* < 0.001, the Mann-Whitney *U* test was applied to calculate *p* values. Scale bars, 5 μm. (E and F) Immunostaining of TEX11 (green) and SYCP3 (red) on zygotene and early-pachytene spermatocyte chromosome spreads of control and *Meiob*^cKO^ mice (E). The number of TEX11 foci was counted (F). “n” represents the number of spermatocytes from 3 mice for each genotype. The data are shown as mean ± S.D. ∗∗∗*p* < 0.001, The Mann-Whitney U test was applied to calculate *p* values. Scale bars, 5 μm.

Stable accumulation of RNF212 is often associated with normal MSH4 loading. Compared with controls, significantly fewer MSH4 foci were observed on the meiotic chromosomes in both zygotene-like and early pachytene-like *Meiob*^cKO^ spermatocytes (Figures 5C and 5D). Likewise, TEX11 recruitment to meiotic chromosomes was significantly impaired in *Meiob*^cKO^ early pachytene spermatocytes (Figures 5E and 5F). Consequently, crossover formation was markedly compromised at mid-pachynema, resulting in significantly fewer MLH1 foci compared to the controls (Figure S7). Taken together, these findings suggest that the MEIOB plays an important role in stabilizing crossover-associated recombination factors and promoting crossover formation, possibly through its cooperation with SPO16.

## Discussion

Two seminal studies unveiled that the ssDNA-binding protein MEIOB plays a pivotal role in the regulation of the recruitment of meiotic recombinases to chromosome axes for DSB repair and recombination.^14^^,^^15^ Later, the potential effect of MEIOB on crossover formation in mid-late pachytene spermatocytes was shown in mouse lines displaying a destabilized MEIOB protein.^20^ In this study, we generated a tamoxifen-inducible *Meiob* conditional knockout (*Meiob*^cKO^) mouse line and revealed that deletion of MEIOB in early pachytene spermatocytes caused premature SC disassembly, which resulted in an arrest prior to the mid-pachytene stage. Further analyses demonstrated both an *in vitro* association and an *in vivo* phenotypic correlation between MEIOB and SPO16. Therefore, the MEIOB-SPO16 interaction may play a crucial role in maintaining the SC structural integrity to promote crossover formation in pachytene spermatocytes.

In our *Meiob*^cKO^ mice, chromosomal synapsis defects were observed, accompanied by dispersed TEX12 signals and markedly reduced TEX11 foci. This reduction in TEX11 signals appears more severe than that reported in constitutive *Meiob* knockout mice,^14^ likely reflecting differences in antibody sensitivity and knockout strategies. Notably, this observation aligns with a previous study reporting dosage-sensitive recombination defects caused by a hypomorphic *Meiob* missense mutation.^20^ In yeast, the interaction between the SC central element protein Ecm11 and Zip4 (the ortholog of mammalian TEX11) acts as a physical connection between crossover formation and SC assembly, and this interaction may be evolutionarily conserved in mammals.^10^^,^^28^ Our findings suggest that MEIOB plays an important role in maintaining proper TEX11 and TEX12 localization during meiosis, which may contribute to crossover formation and SC integrity.

In mice, premature desynapsis during pachynema typically results in defects in the transition from Prometaphase I to Metaphase I.^29^^,^^30^^,^^31^^,^^32^ *Meiob*^cKO^ spermatocytes exhibited premature desynapsis at the early pachytene stage and failed to progress to mid-pachytene, likely due to a compromised recombination function without MEIOB. However, spermatocytes with premature SC disassembly from both *Spo16*^−/−^ and *Meiob*^cKO^ mice exhibited similar disappearance of HORMAD1 and BRCA1 proteins from autosomal axes, thereby confirming the observed phenotype in the present study is indeed premature desynapsis. Nevertheless, we cannot completely exclude the possibility that a subtle defect in SC assembly contributes to the observed phenotypes, given the close relationship between SC assembly and maintenance. Further investigation, including detailed time-course analyses in a synchronized mouse model, will be required to distinguish these possibilities.

Two research groups have independently reported in the budding yeast that the physical interaction between dHJs and ZMM proteins is crucial for maintaining SC stability in order to coordinate meiotic progression and ensure crossover formation.^7^^,^^8^ In this study, we uncovered the pivotal role of MEIOB in preserving the structural integrity of the SC, which is likely mediated by the interaction between MEIOB and SPO16, a key member of the ZMM protein family. This finding implies that the interaction mechanism between dHJs and ZMM proteins in yeast may be conserved in mice. SPO16 is a highly conserved protein involved in stabilizing early recombination intermediates and SC in yeast, *Arabidopsis*, and mice.^11^^,^^12^^,^^33^^,^^34^ Although MEIOB does not directly interact with TEX11, our findings confirm a functional interdependence between MEIOB and SPO16. We propose that the functional association between MEIOB and SPO16 supports the efficient recruitment of TEX11 and subsequent crossover formation (Figure 6).

**Figure 6:**
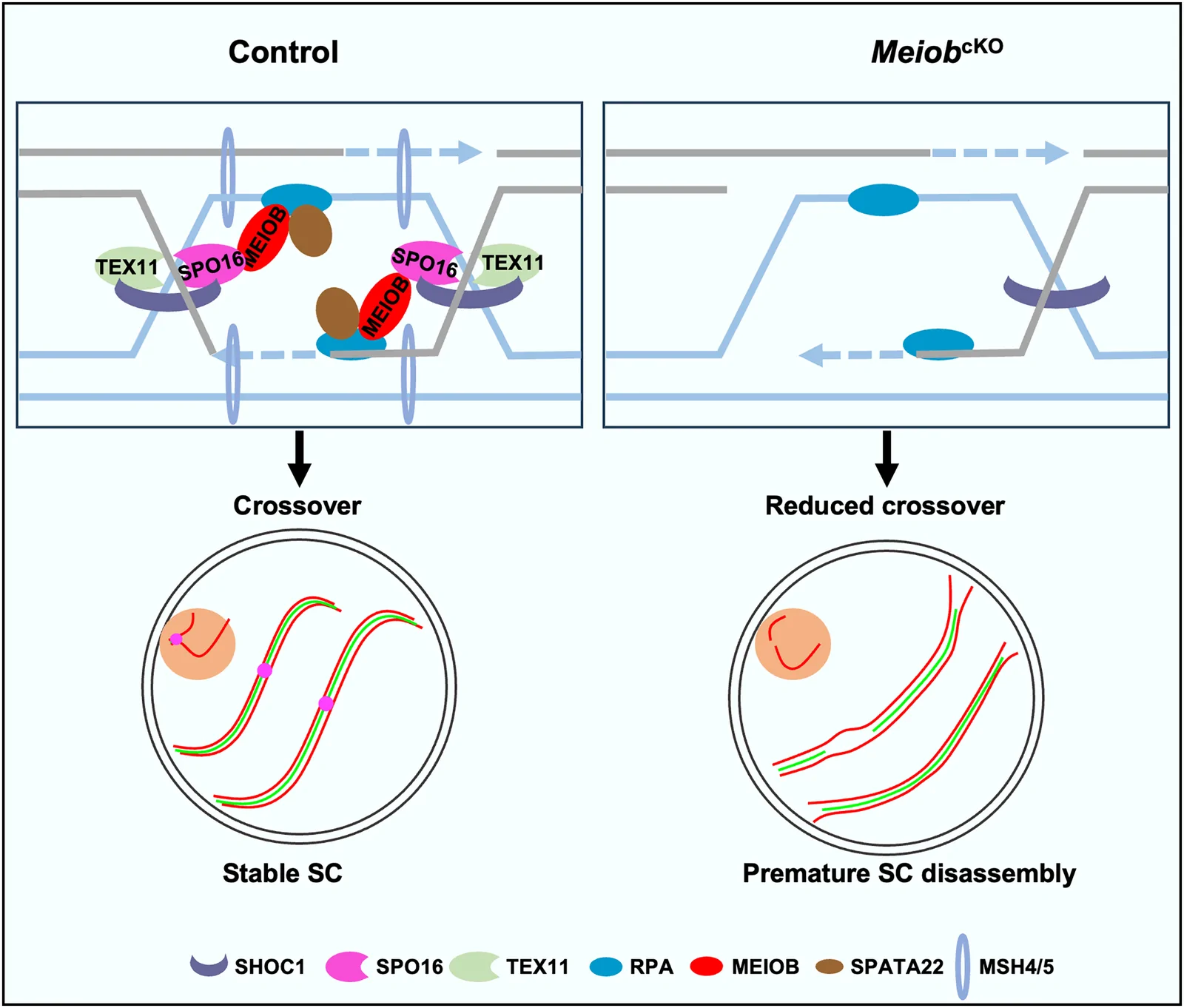
Schematic illustration of MEIOB-SPO16 interaction in the regulation of SC structural stability and crossover formation during male meiosis The MEIOB-SPATA22 complex binds to ssDNA via an interaction with RPA, while the MEIOB interacts with SPO16 to promote the recruitment of TEX11 to recombination intermediates, thereby enhancing the formation of dHJs. In early pachytene spermatocytes, the absence of MEIOB leads to reduced ZZS complex stability and a failure to maintain SC structure. Ultimately, this results in the premature disassembly of the SC and a significant reduction in crossovers.

In summary, our results uncover a role of MEIOB in sustaining SC integrity and promoting meiotic crossover formation. This function may be mediated by a direct MEIOB-SPO16 interaction. Therefore, we propose that MEIOB may serve as a bridge mediating the association between HJs and the ZZS complex, thereby promoting crossover formation and maintaining SC structural integrity. A deeper understanding of how MEIOB interacts with other meiotic proteins and functions within specific molecular mechanisms will provide further insights into the regulation of meiotic recombination.

### Limitations of the study

While our findings highlight the essential role of MEIOB in sustaining SC stability during meiosis, characterizing the precise *in vivo* localization and dynamics of endogenous SPO16 remains constrained by the absence of high-quality specific antibodies. Additionally, although the MEIOB-SPO16 interaction was validated via *in vitro* and electroporation approaches, the exact molecular mechanisms and structural details of their interplay with other meiotic factors under physiological conditions warrant further investigation. Finally, this study was limited to male meiosis, and whether MEIOB similarly cooperates with SPO16 to sustain SC integrity during female meiosis remains to be established.

## Resource availability

### Lead contact

Further information and requests for the resources and reagents should be directed to and will be fulfilled by the Lead Contact, Dongteng Liu (liudt@hku.hk).

### Materials availability

All unique materials generated in this study will be made available upon request to the lead contact.

### Data and code availability

• Data: Data reported in this paper will be shared by the lead contact upon request.
• Code: This study does not include newly generated code.
• Additional information: Any supporting documentation required to repeat data reported in this paper is available from the lead contact upon request.

## Acknowledgments

This study was supported by grants from the Shenzhen-Hong Kong-Macau Type C Project (SGDX20230821091501011), the Shenzhen Science and Technology Program, China (No. KQTD20190929172749226), the 10.13039/501100012151Sanming Project of Medicine in Shenzhen (SZSM202211009), the Shanghai Pujiang Program (No. 23PJ1410300), the Faculty Development Grants from Hubei University of Medicine (2025QDJZR01), and the 10.13039/501100017607Shenzhen Fundamental Research Program (grant no. JCYJ20220530154805013). We thank the University of Hong Kong-Shenzhen Hospital (HKU-SZH) Core Laboratory for providing technical support for confocal microscopy.

## Author contributions

H.F.: conceptualization, Study design; methodology; investigation; writing—original draft, writing—review and editing. B.J.: methodology; investigation; writing—original draft. Y.G.: methodology; investigation; writing—review and editing. M.Z.: writing—review and editing. L.H.: investigation. H.W.: investigation. X.W.: investigation. M.L.: investigation; funding acquisition. M.L.: methodology; resources. J.S.: funding acquisition; writing—review and editing. L.H.: supervision, funding acquisition, writing—review and editing. D.L.: conceptualization, Study design, supervision, investigation, funding acquisition, writing—original draft, writing—review and editing.

## Declaration of interests

The authors declare no competing interests.

## STAR★Methods

### Key resources table

**Table:** 

REAGENT or RESOURCE	SOURCE	IDENTIFIER
**Antibodies**		

Mouse anti-γH2AX (IF, 1:500)	Millipore	Cat# 05–636; RRID: AB_309864
Mouse anti-SYCP3 (IF, 1:200)	Abcam	Cat# ab97672; RRID: AB_10678841
Rat anti-SYCP3 (IF, 1:200)	This paper	N/A
Rabbit anti-SYCP1 (IF, 1:200)	Abcam	Cat# ab15090; RRID: AB_301636
Rat anti-H1T (IF, 1:400)	Liu et al.^35^	N/A
Rat anti-MEIOB (IF, 1:200)	This paper	N/A
Rabbit anti-SPATA22 (IF, 1:200)	Proteintech	Cat# 16989-1-AP; RRID: AB_2286640
Rabbit anti-TEX11 (IF, 1:200)	provided by Mengcheng Luo	N/A
Rabbit anti-RNF212 (IF, 1:100)	provided by Mengcheng Luo	N/A
Rabbit anti-MSH4 (IF, 1:50)	provided by Mengcheng Luo	N/A
Rabbit anti-HORMAD1 (IF, 1:400)	Proteintech	Cat# 13917-1-AP; RRID: AB_2120844
Mouse anti-BRCA1 (IF, 1:100)	Santa Cruz	Cat# sc-135732; RRID: AB_2243740
Rabbit anti-TEX12 (IF, 1:200)	Proteintech	Cat# 17068-1-AP; RRID: AB_2201875
Rabbit anti-SIX6OS1 (IF, 1:300)	Liu et al.^35^	N/A
Mouse anti-MLH1 (IF, 1:50)	BD Biosciences	Cat# 551092; RRID: AB_394041
Rabbit anti-Cleaved PARP (Asp214) (IF, 1:150)	Cell Signaling Technology	Cat# 94885; RRID: AB_2800237
Mouse anti-FLAG (IF, 1:200)	Sigma	Cat# F3165; RRID: AB_259529
Rabbit anti-β-actin (WB, 1:5,000)	Abcam	Cat# ab179467; RRID: AB_2737344
Rabbit anti-HA (WB, 1:2,000)	Proteintech	Cat# 51064-2-AP; RRID: AB_11042321
Goat anti-Rat IgG H&L (Alexa Fluor 488) (IF, 1:200)	Abcam	Cat# ab150165; RRID: AB_2650997
Goat anti-Rat IgG H&L (Alexa Fluor 594) (IF, 1:200)	Abcam	Cat# ab150160; RRID: AB_2756445
Goat anti-Mouse IgG H&L (Alexa Fluor 488) (IF, 1:200)	Abcam	Cat# ab150113; RRID: AB_2576208
Goat anti-Mouse IgG H&L (Alexa Fluor 594) (IF, 1:200)	Abcam	Cat# ab150120; RRID: AB_2631447
Goat anti-Rabbit IgG H&L (Alexa Fluor 488) (IF, 1:200)	Abcam	Cat# ab150081; RRID: AB_2734747
Goat anti-Rabbit IgG H&L (Alexa Fluor 594) (IF, 1:200)	Abcam	Cat# ab150084; RRID: AB_2734147
Goat anti-Rabbit IgG (H + L)-HRP Conjugate (WB, 1:5,000)	Bio-Rad	Cat# 1706515; RRID: AB_2617112
Goat anti-Mouse IgG (H + L)-HRP Conjugate (WB, 1:5,000)	Invitrogen	Cat# 31430; RRID: AB_228307

**Chemicals, peptides, and recombinant proteins**		

DNase I	Applichem	Cat# A3778
Complete Protease Inhibitor	Roche	Cat# 04693159001
LipofectamineTM 3000 Transfection Reagent	Invitrogen	Cat# L3000008
Triton X-100	Sigma-Aldrich	Cat# T9284
Bouin’s solution	Sigma-Aldrich	Cat# HT10132
Fetal Bovine Serum	Gibco	Cat# 16000044
Penicillin-Streptomycin	Gibco	Cat# 15140122
PMSF Protease Inhibitor	Thermo Fisher Scientific	Cat# 36978

**Experimental models: Cell lines**		

Human: HEK293T cells	ATCC	Cat# CRL-3216 RRID: CVCL_0063

**Experimental models: Organisms/strains**		

Mouse: *Spo16*^−/−^	Liu et al.^12^	N/A
Mouse: *Meiob*^fl/fl^	This paper	N/A
Mouse: *Ddx4*-Cre^ERT2^	Liu et al.^35^	N/A

**Oligonucleotides**		

sgRNAs and primers used in this study are listed in Table S1	This paper	N/A

**Recombinant DNA**		

pCAG-GFP	Addgene	RRID: Addgene_11150
pcDNA3.1-3×HA-Meiob-GFP	This paper	N/A
pcDNA3.1-3×FLAG-Shoc1-GFP	This paper	N/A
pcDNA3.1-3×FLAG-Tex11-GFP	This paper	N/A
pCAG-3×FLAG-Spo16-GFP	This paper	N/A

**Software and algorithms**		

GraphPad Prism	GraphPad	RRID: SCR_002798

### Experimental model and study participant details

#### Mouse strains

The *Spo16* knockout mouse model was generated using CRISPR/Cas9-mediated genome editing as previously described.^12^ The *Meiob*-floxed mice were produced by Cyagen Biosciences (Suzhou, China) using the CRISPR/Cas9 technology. In order to generate a 5.6-kb DNA deletion, the guide RNAs were designed to insert *loxP* sites at the 6^th^ and 10^th^ introns. *Ddx4*-Cre^ERT2^ mice were generated as described previously.^35^ All mouse lines used in this study, including *Meiob*^fl/fl^, *Ddx4*-Cre^ERT2^, *Spo16*^−/−^, and the wild-type mice used for testicular electroporation, were maintained on a C57BL/6 background, and only male mice were analyzed.

Tamoxifen (T5648; Sigma-Aldrich, Burlington, MA, USA) was dissolved in corn oil and heated at 55°C in a shaking incubator for 1 h until fully dissolved, to achieve a final concentration of 20 mg/mL. To induce germ cell-specific *Meiob* gene deletion, tamoxifen (80 mg/kg body weight) was intraperitoneally injected into *Meiob*^fl/fl^; *Ddx4*-Cre^ERT2^ male mice, as well as *Meiob*^+/+^; *Ddx4-*Cre^ERT2^ or *Meiob*^fl/+^ pups, starting at postnatal day 14 (PD14). The injections were performed daily for 4 consecutive days. Testicular samples were collected at various time points after treatment completion for subsequent analyses.

The mice were maintained under specific pathogen-free (SPF) conditions in a temperature-controlled environment (20°C–22°C) with a 12-h light/dark cycle and 50–70% relative humidity. All animal procedures were approved by the Ethics Committee of Shenzhen Hospital of the University of Hong Kong (No. hkuszh2020031) and was conducted in accordance with the guidelines of the committee. The genotyping primers and sequences of mouse gRNAs used are listed in Table S1. The PCR program was as follows: an initial denaturation at 94°C for 3 min, followed by 30 cycles of denaturation at 94°C for 20 s, annealing at 62°C for 20 s, and extension at 72°C for 30 s, and a final extension at 72°C for 5 min.

#### Cell culture and transfections

HEK293T (ATCC, CRL-3216, USA) cells were authenticated by ATCC and tested negative for mycoplasma contamination. HEK293T cells were cultured in DMEM (Dulbecco’s Modified Eagle Medium) supplemented with 10% fetal bovine serum (FBS) (GIBCO, 16000044, USA). For cell digestion and passaging, cells were incubated with 0.25% trypsin-EDTA at 37°C for 3 min. To terminate trypsinization, an equal volume of DMEM containing 10% FBS was added. The cells were then centrifuged at 300 × g for 5 min and the supernatant was discarded. The cell pellet was resuspended in fresh medium and passaged at an appropriate dilution ratio. Transfection was performed when the cells reached 70–80% confluence.

For transfection, 5 μL of Lipofectamine 3000 (Invitrogen, L3000008, USA) reagent was diluted in 125 μL Opti-MEM medium and mixed thoroughly. Separately, 2.5 μg of plasmid DNA was diluted in 125 μL of Opti-MEM. The plasmid solution was then combined with diluted Lipofectamine 3000 reagent, and 5 μL of P3000 reagent was added to the mixture, followed by thorough mixing. The resulting DNA-Lipofectamine complexes were incubated at room temperature for 10–15 min. After incubation, the complex was gently added to the culture medium, and the medium was mixed by gentle swirling to ensure even distribution. Subsequent experiments were conducted 48 h post transfection.

### Method details

#### Histological analysis

After euthanizing the mice, the testes were immediately excised and fixed in Bouin’s solution at room temperature overnight. Following fixation, tissues were dehydrated using a series of graded ethanol solutions and embedded in paraffin. Tissue sections (5 μm thick) were obtained using a microtome. After deparaffinization with xylene and rehydration through a graded ethanol series, the sections were stained with haematoxylin for histological examination. Images were captured using an inverted microscope (Zeiss, Oberkochen, Germany).

#### Spermatocyte surface nuclear spread analyses and immunofluorescence

Spermatocyte chromosome spreads were prepared as previously described.^16^^,^^36^ Fresh testicular tissue samples were washed with phosphate-buffered saline (PBS) to remove tunica albuginea and subsequently subjected to hypotonic treatment in HEB buffer [30 mM tris-HCl, 17 mM trisodium citrate dihydrate, 50 mM sucrose, 5 mM EDTA, 0.5 mM dithiothreitol (DTT), and 1 mM phenylmethylsulfonyl fluoride (PMSF)] for 30 min. The seminiferous tubules were then mechanically dissociated in a 0.1 M sucrose solution using a pair of fine forceps. Approximately 10 μL of the resultant suspension was placed on slides pre-coated with fixative buffer [1% paraformaldehyde, and 0.15% Triton X-100 (pH 9.2)] to allow for further hypotonic spreading. The preparations were air-dried at room temperature for 1 h before being subjected to immunofluorescence staining or stored at −80°C.

For immunofluorescence staining, slides were washed with PBST (PBS with 0.05% Triton X-100) for 5 min. Following this, incubation with primary and secondary antibodies was conducted, and the specifics of the antibodies used are detailed in key resources table. Fluorescence signals were captured using an LSM 900 confocal microscope (Zeiss, Oberkochen, Germany).

#### Immunoprecipitation and immunoblotting assays

Full-length cDNAs encoding meiotic proteins MEIOB, SPO16, SHOC1, and TEX11 were amplified by RT-PCR using murine testis RNA as template. For expression constructs, the *Shoc1*, and *Tex11* cDNAs were cloned into pcDNA3.1 to generate N-terminal 3×FLAG-tagged and C-terminal GFP-fusion proteins. Similarly, the *Spo16* cDNAs was cloned into pCAG to generate N-terminal 3×FLAG-tagged and C-terminal GFP-fusion protein. And the *Meiob* cDNA was cloned into pcDNA3.1 to create an N-terminal 3×HA-tagged and C-terminal GFP-fusion protein. Pipette 500 μL of IP Lysis Buffer (supplemented with 1 μM PMSF and protease inhibitor cocktail tablet: 1 tablet per 10 mL of lysis buffer) was added to the cell precipitate, mixed thoroughly, placed on ice for 40 min, and vortexed every 5 min for 8 s. After pre-cooling the centrifuge to 4°C, the lysed sample was centrifuged at 12,000 g for 15 min and the supernatant was transferred to a new 1.5 mL Eppendorf tube. Next, for each sample, 20 μL of HA/FLAG magnetic beads was pipetted into a new tube, the liquid was aspirated on a magnetic rack, washed three times with diluted and pre-cooled 0.75% TBST, and then resuspended in IP Lysis Buffer. Beads (20 μL) were dispensed into each sample tube, mixed thoroughly, and incubated overnight at 4°C. Finally, the sample protein solution was aspirated on the magnetic rack, the beads were washed three times with diluted and pre-cooled TBST, and 50 μL of 1 × dual-colour loading buffer was added to each sample, boiled at 95°C for 5 min, and stored at −20°C for at least one year or used directly for immunoblotting experiments.

The samples were boiled before SDS-PAGE analysis. SDS-PAGE was performed at 120 voltage for 60 min, followed by transfer to a PVDF membrane under constant current conditions in an ice box (250 mA for 2.5 h). The membrane was blocked with 5% skim milk-TBST at RT for 1 h and incubated with the primary antibody at 4°C overnight. After rinsing, the membranes were incubated with an HRP-conjugated secondary antibody at RT for 2 h. Finally, the membrane was rinsed again and exposed to ECL solution in a darkroom for imaging, with exposure time adjusted based on signal strength.

#### Mouse testicular tissue electroporation

Exogenous expression of *Spo16* cDNAs in the testes was performed as previously described.^12^ Briefly, the *Spo16* cDNA was cloned into the pCAG-GFP vector (Addgene #11150). Plasmid DNA (5 μg/μL) was mixed with 1 μL Fast Green and 9 μL plasmid solution and injected into the testes of postnatal day 20 (PD20) mice. Electroporation was performed using a specific electrical pulse protocol. Two days post-electroporation, testis tissues were harvested for spermatocyte chromosome spreading and immunofluorescence analysis to detect GFP signals.

### Quantification and statistical analysis

All statistical analyses were performed and graphs were generated using the GraphPad Prism 9 software. Data are presented as the mean ± S.D. All statistical tests employed are detailed in the figure legends or STAR Methods section. No human subjects or samples were used in this study. Statistical significance is indicated as follows: ns, not significant; ∗*p* < 0.05, ∗∗*p* < 0.01, and ∗∗∗*p* < 0.001.
