# Supplementary material for: The ssDNA-binding protein MEIOB sustains synaptonemal complex integrity by stabilizing recombination intermediates during male meiosis
Source: iScience. 2026 Nov 2;29(11):118103. doi: 10.1016/j.isci.2026.118103 (PMC13755600; doi:10.1016/j.isci.2026.118103)
Supplement: Supplementary file 1 [file mmc1.pdf]

iScience, Volume *29*

Supplemental information

The ssDNA-binding protein MEIOB sustains synaptonemal complex integrity by stabilizing recombination intermediates during male meiosis

Haiwei Feng, Binjie Jiang, Yanling Gao, Muhammad Zubair, Li Hu, Hongling Wang, Xuemei Wang, Miao Li, Mengcheng Luo, Jingxia Sun, Lijun Huo, and Dongteng Liu

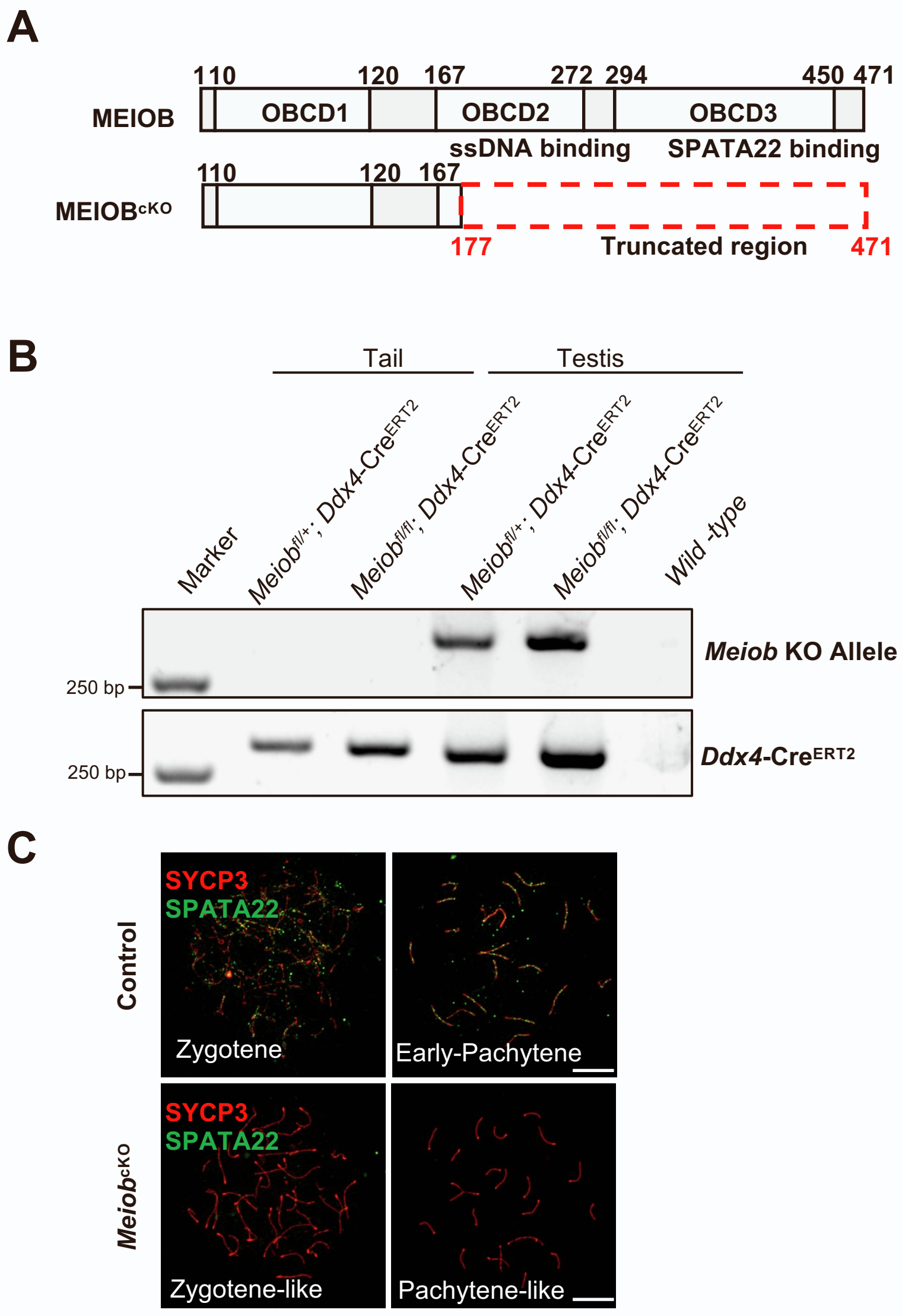

Figure S1. Genotyping and immunofluorescence confirmation of *Meiob*^{cKO} Mice.

(A) Schematic diagram of MEIOB and MEIOB^{cKO} protein structures. The MEIOB protein is composed of 471 amino acids, including 3 OB-fold. Deletion of residues 177–471 in the MEIOB^{cKO} allele produces a truncated protein composed of the N-terminal OB domain. (B) Genotyping was performed on mouse tail and testis tissue harvested from tamoxifen-induced *Meiob*$^{fl/fl}$; *Ddx4*-Cre^{ERT2} and control littermates. The position of the 250 bp size marker is indicated to the left of each gel. (C) Immunostaining of SPATA22 (green) and SYCP3 (red) on spermatocytes chromosome spreads of control and *Meiob*^{cKO} mice. Scale bars, 5 μm.

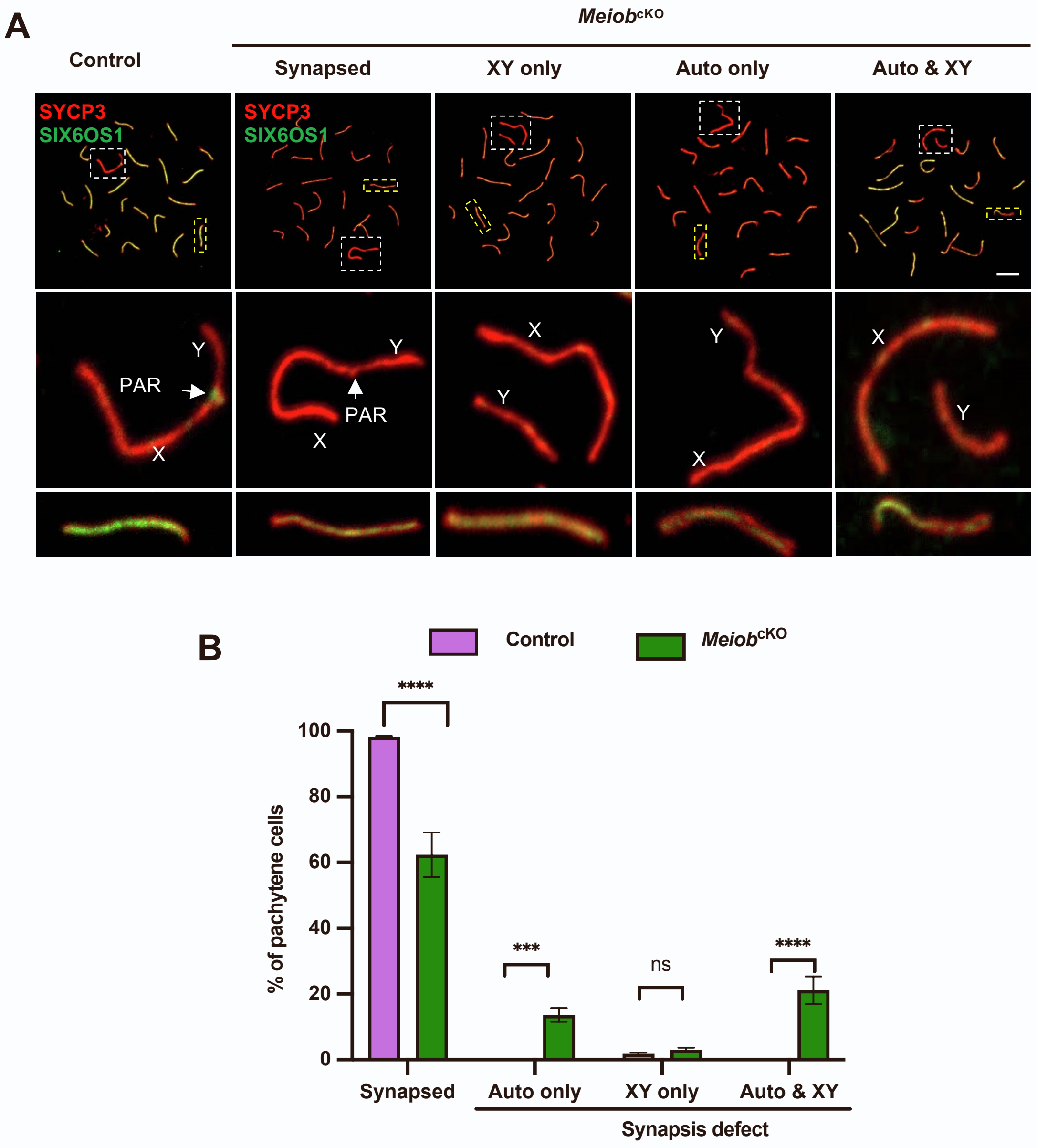

Figure S2. ***Meiob*^{cKO} pachytene spermatocytes sex chromosome synaptic defects.**

(A) Immunostaining of SIX6OS1 (green) and SYCP3 (red) on spermatocytes chromosome spreads of control and *Meiob*^{cKO} mice. Based on synapsis defects, *Meiob*^{cKO} pachytene spermatocytes were classified into four groups: (1) fully synapsed chromosomes, (2) synapsis defects restricted to sex chromosomes, (3) synapsis defects limited to autosomes, and (4) synapsis defects involving both autosomes and sex chromosomes. A magnified view of the areas within the yellow and white boxes shows an autosomes and the XY chromosomes, respectively. Scale bar, 5 µm. (B) Quantitative analysis of synapsis defects in pachytene spermatocytes from control and *Meiob*^{cKO} mice. "Auto only" indicates autosome synapsis defects; "XY only" indicates sex chromosome synapsis defects; "Auto & XY" indicates defects in both autosomes and sex chromosomes. A total of 524 pachytene spermatocytes from 3 mice per genotype were analyzed. The data are shown as mean ± S.D. Students' t test, ns, not significant, ***$P < 0.001$. ****$P < 0.0001$.

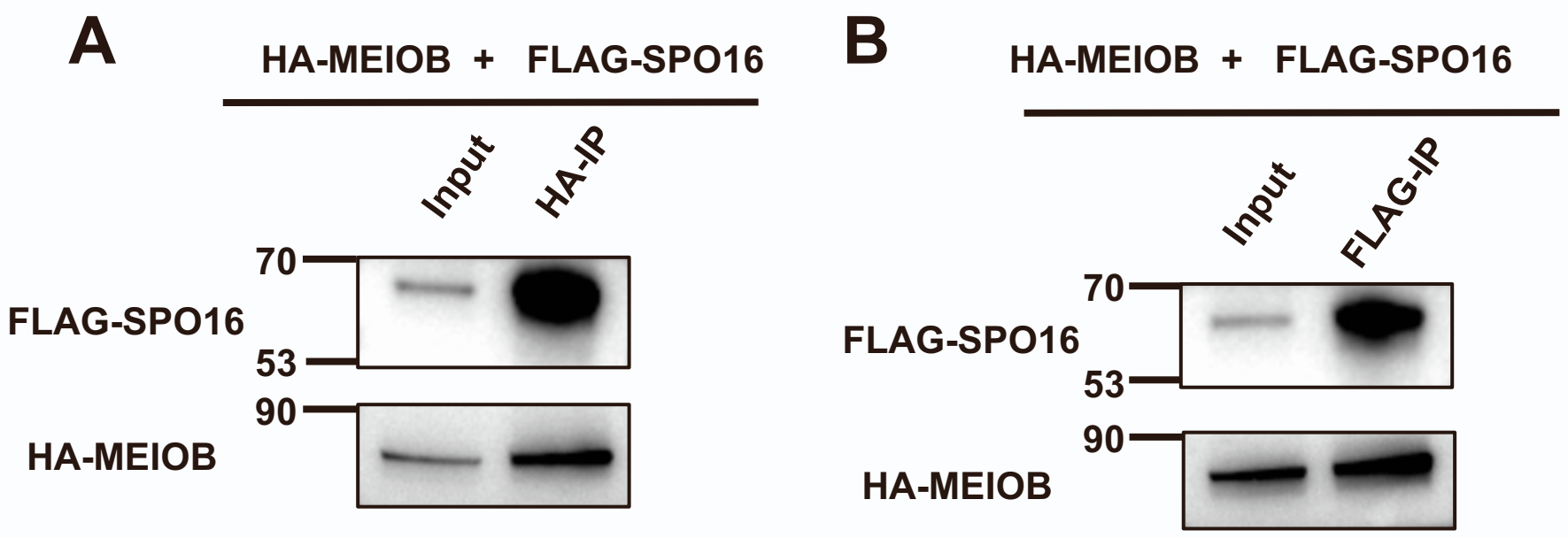

Figure S3. DNA-independent interaction between MEIOB and SPO16.

Reciprocal Co-IP assays demonstrate the physical interaction between HA-MEIOB and FLAG-SPO16 in HEK293T cells. Cell lysates were treated with DNase I prior to immunoprecipitation to eliminate DNA-dependent indirect binding. (A) HA-MEIOB pulls down FLAG-SPO16. (B) Conversely, FLAG-SPO16 efficiently pulls down HA-MEIOB.

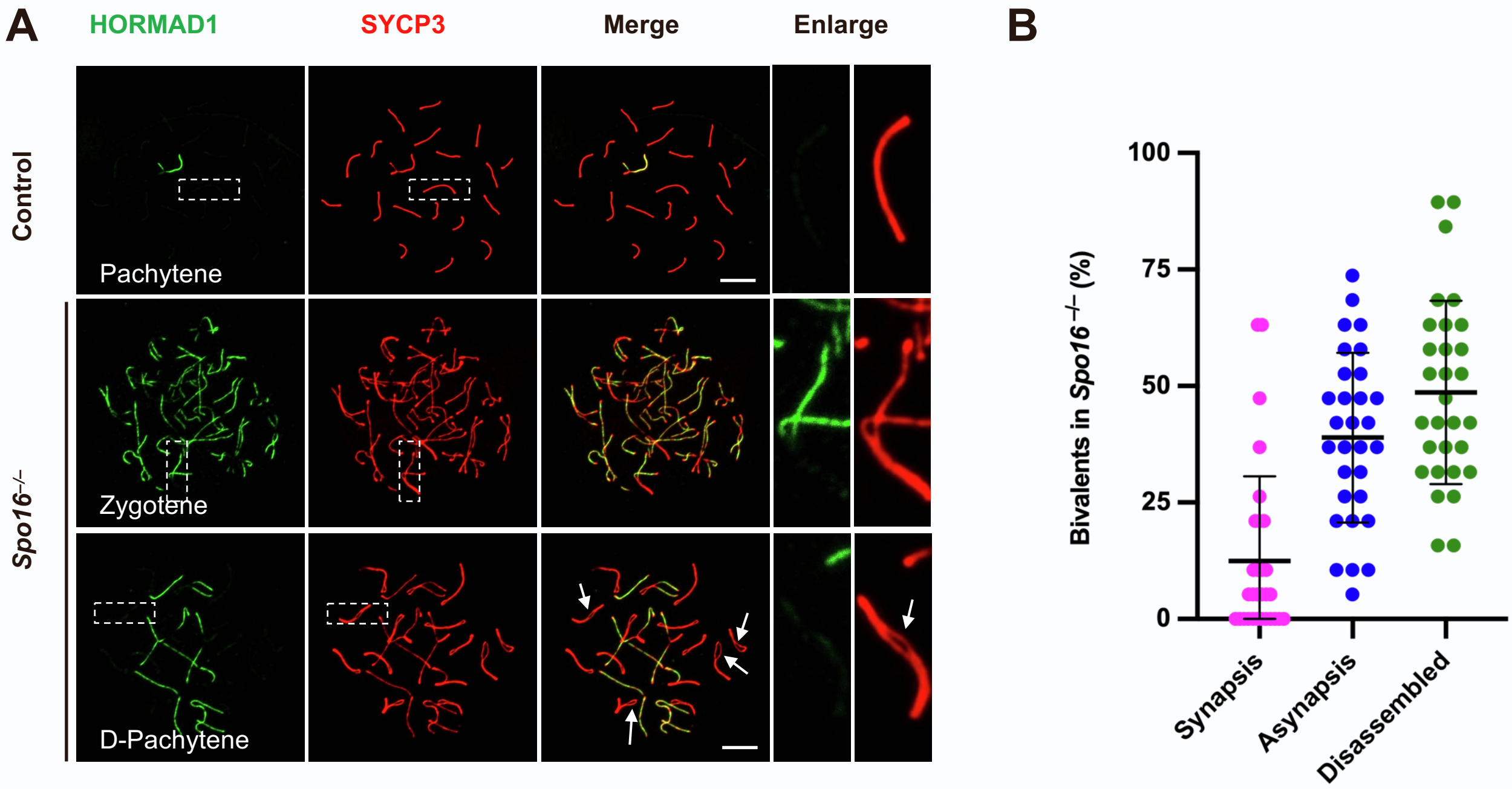

Figure S4. Prematurely disassembled chromosomes were HORMAD1-negative in *Spo16*$^{-/-}$ mice.

(A) Immunostaining of HORMAD1 (green) and SYCP3 (red) on spermatocytes chromosome spreads of control and *Spo16*$^{-/-}$ mice. A magnified view of the areas within the white boxes shows an autosome bivalent. White arrows indicate prematurely disassembled synaptonemal complex. Scale bars, 5 µm. (B) Frequency of synaptic, asynaptic and disassembled chromosomes in pachytene spermatocytes of *Spo16*$^{-/-}$ mice. A total of 30 pachytene spermatocytes were analyzed from two *Spo16*$^{-/-}$ animals. The data are shown as mean ± S.D.

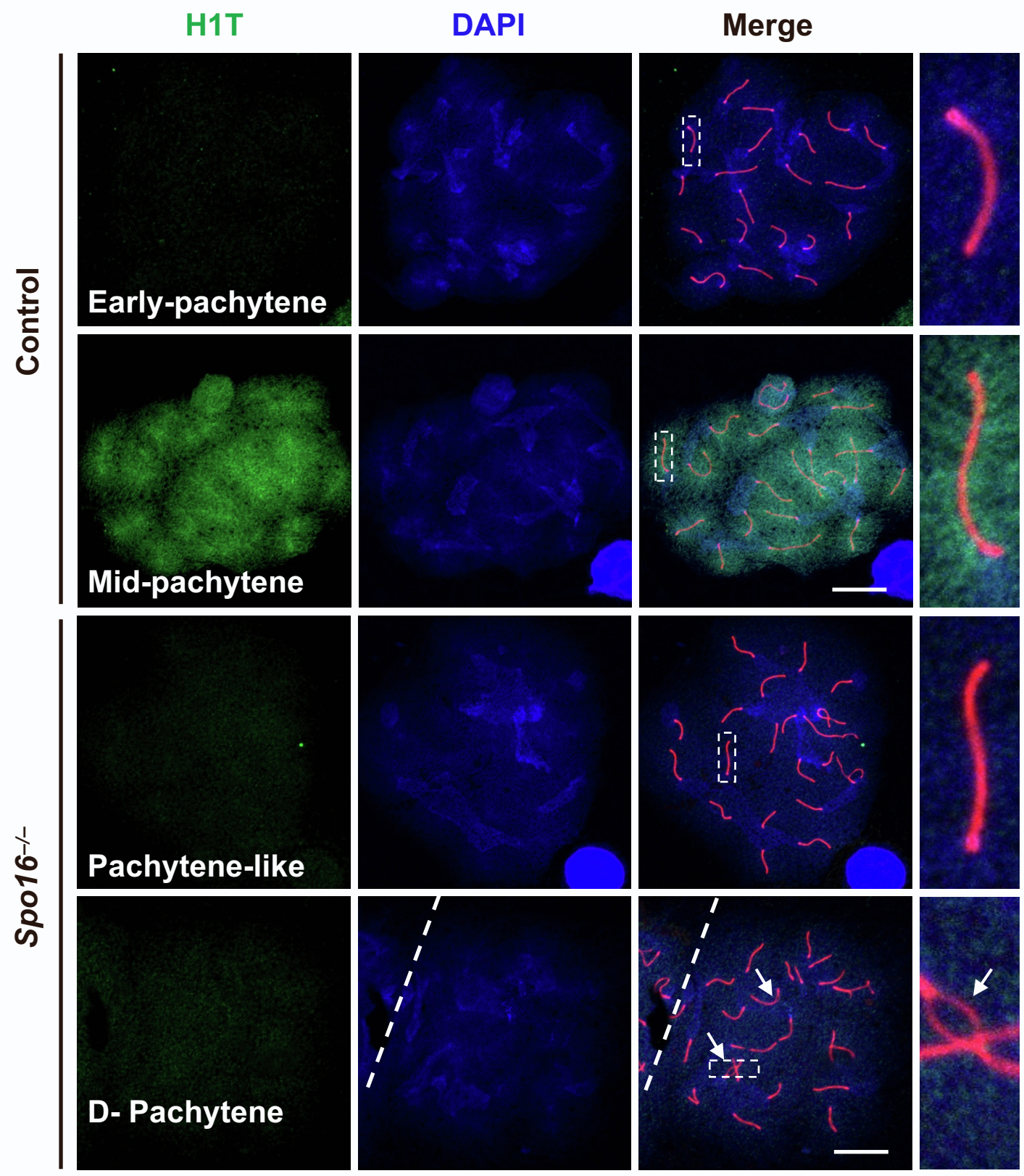

Figure S5. Both pachytene-like spermatocytes and prematurely disassembled spermatocytes were H1T-negative in *Spo16*$^{-/-}$ mice.

Immunostaining of H1t (green) and SYCP3 (red) on spermatocytes chromosome spreads of control and *Spo16*$^{-/-}$ mice. A magnified view of the areas within the white boxes shows an autosome bivalent. White arrows indicate prematurely disassembled synaptonemal complex. Scale bars, 5 µm.

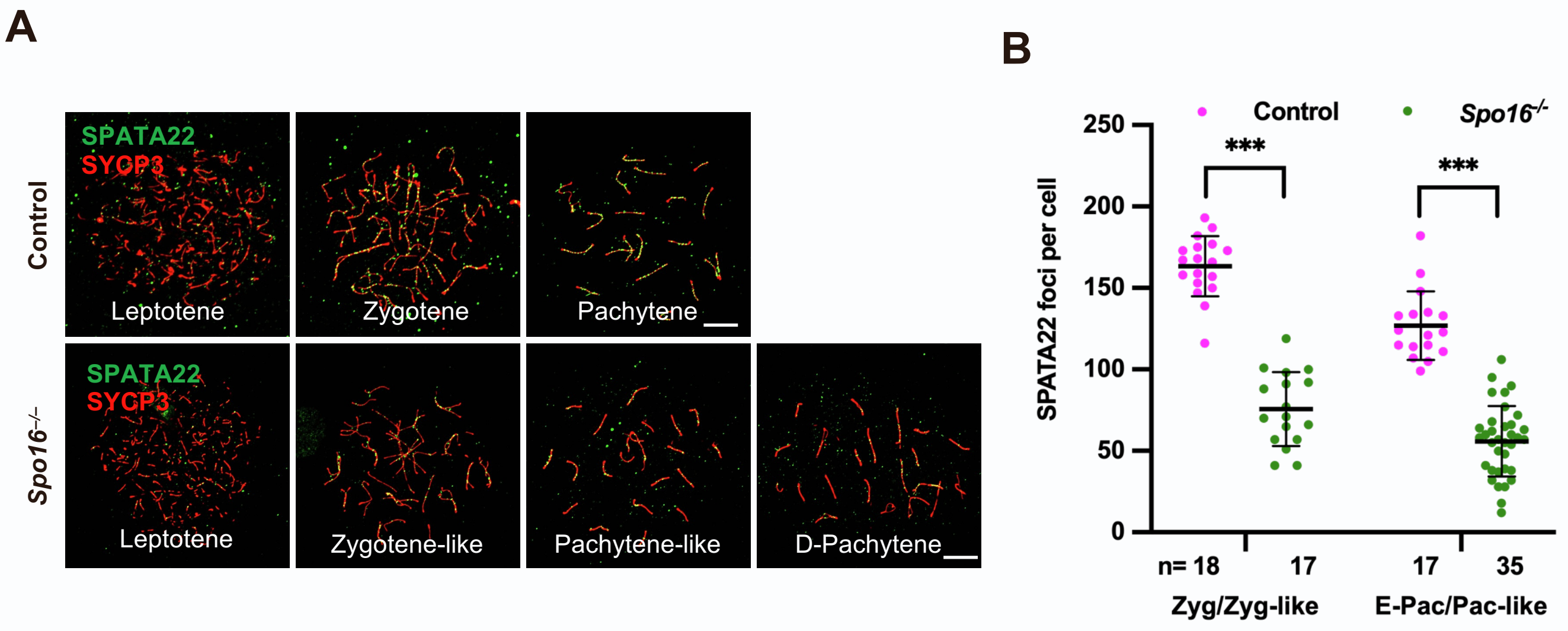

Figure S6. SPO16 is essential for the stability of the SPATA22 on chromosome axes during meiotic prophase I. (A) Immunostaining of SPATA22 (green) and SYCP3 (red) on pachytene spermatocytes chromosome spreads of control and *Spo16*$^{-/-}$ mice (A). The number of SPATA22 foci was counted (B). "n" represents the number of spermatocytes analyzed from 3 mice per genotype. Scale bars, 5 µm. The data are shown as mean ± S.D. *** $P < 0.001$. The Mann–Whitney U test was applied to calculate P values.

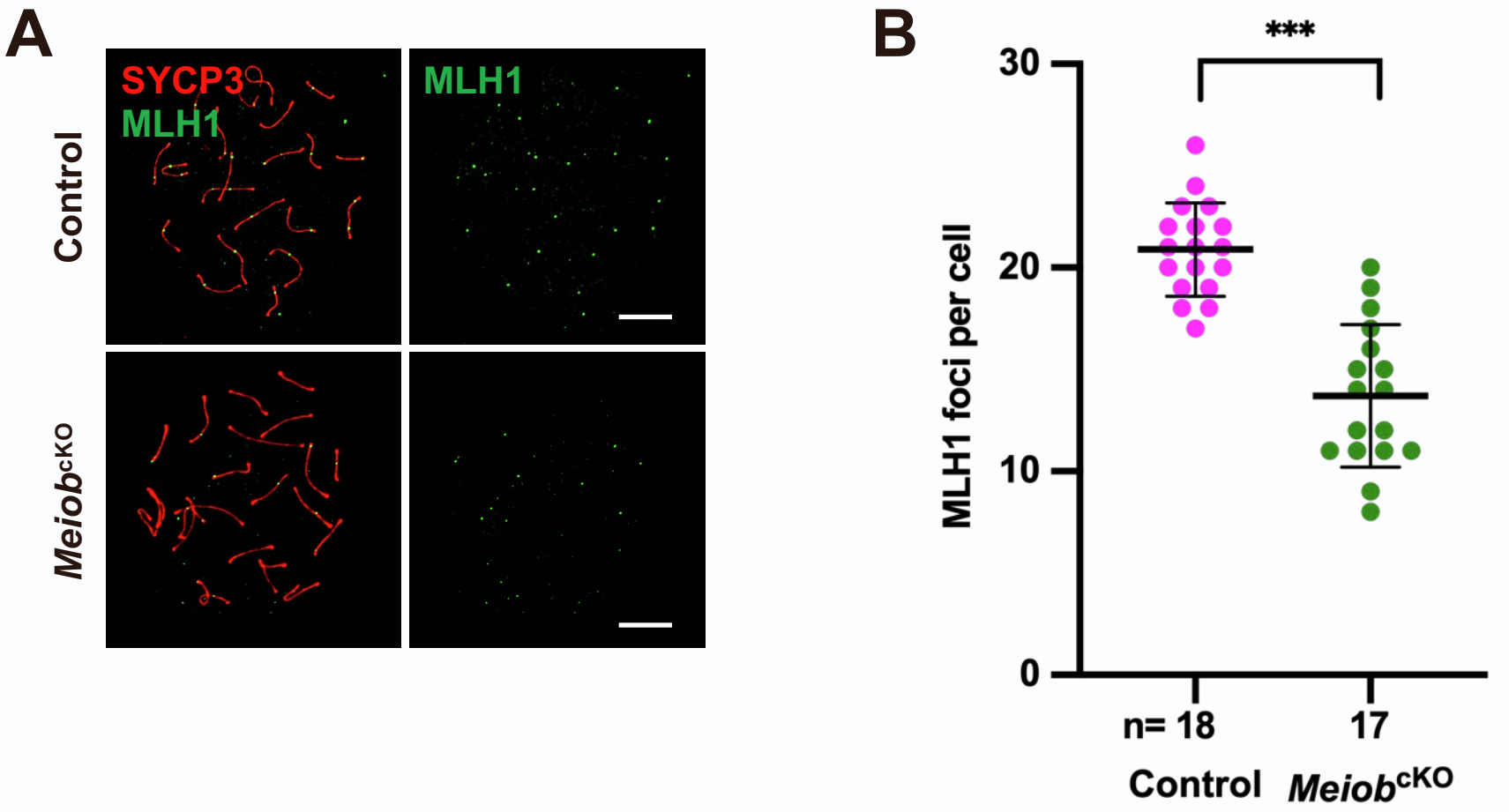

Figure S7. *Meiob*^{cKO} pachytene spermatocytes exhibited defects in crossover formation.

(A) Immunostaining of MLH1 (green) and SYCP3 (red) on mid-late pachytene spermatocytes chromosome spreads of PD21 control and *Meiob*^{cKO} mice. Scale bars, 5 μm. (B) Comparison of MLH1 foci number between control and *Meiob*^{cKO} mid-late pachytene spermatocytes. “n” represents the number of spermatocytes analysed from 3 mice per genotype. The data are shown as mean ± S.D. *** $P < 0.001$. The Mann–Whitney U test was applied to calculate P values.
